# CAR-Macrophages and CAR-T Cells Synergistically Kill Tumor Cells In Vitro

**DOI:** 10.3390/cells11223692

**Published:** 2022-11-21

**Authors:** Maoxuan Liu, Junchen Liu, Ziwei Liang, Kun Dai, Jiangyu Gan, Qi Wang, Yang Xu, Youhai H. Chen, Xiaochun Wan

**Affiliations:** 1 Guangdong Immune Cell Therapy Engineering and Technology Research Center, Center for Protein and Cell-Based Drugs, Institute of Biomedicine and Biotechnology, Shenzhen Institute of Advanced Technology, Chinese Academy of Sciences, Shenzhen 518055, China; 2 College of life Sciences, University of Chinese Academy of Sciences, Beijing 100049, China; 3 School of Medicine, Southern University of Science and Technology, Shenzhen 518055, China

**Keywords:** CAR-M, CAR-T, synergy, immunotherapy, FcRγ

## Abstract

Chimeric antigen receptor (CAR)-expressing macrophages (CAR-M) have a great potential to improve cancer therapy, as shown from several recent preclinical studies. However, unlike CAR-T cell therapy, which has been widely studied, the efficacy and limitations of CAR-M cells remain to be established. To address this issue, in the present study, we compared three intracellular signaling domains (derived from common γ subunit of Fc receptors (FcRγ), multiple EGF-like-domains protein 10 (Megf10), and the CD19 cytoplasmic domain that recruits the p85 subunit of phosphoinositide-3 kinase (PI3K), respectively) for their ability to promote primary CAR-M functions, and investigated the potential synergistic effect between CAR-M and CAR-T cells in their ability to kill tumor cells. We found that CAR-M^FcRγ^ exerted more potent phagocytic and tumor-killing capacity than CAR-M^Megf10^ and CAR-M^PI3K^. CAR-M and CAR-T demonstrated synergistic cytotoxicity against tumor cells in vitro. Mechanistically, the inflammatory factors secreted by CAR-T increased the expression of costimulatory ligands (CD86 and CD80) on CAR-M and augmented the cytotoxicity of CAR-M by inducing macrophage M1 polarization. The upregulated costimulatory ligands may promote the fitness and activation of CAR-T cells in turn, achieving significantly enhanced cytotoxicity. Taken together, our study demonstrated for the first time that CAR-M could synergize with CAR-T cells to kill tumor cells, which provides proof-of-concept for a novel combinational immunotherapy.

## 1. Introduction

Cancer is a leading cause of death worldwide, accounting for around 10 million deaths annually [1]. In recent years, cellular immunotherapy has emerged as a promising approach for treating cancer. Chimeric antigen receptor T (CAR-T) cells have shown clinical efficacy in numerous hematological malignancies, leading to the approval of six CD19 or BCMA-targeted CAR-T products by FDA. Due to the success of CAR-T cell therapy in the clinic, further research has been carried out to engineer other potent CAR immune cells. The CAR has become a promising approach to increase the cancer recognition capacity of immune cells.

Macrophages are innate immune cells that intrinsically possess broad therapeutic effector functions, including direct tumor phagocytosis, active trafficking to tumor sites and activation of the tumor microenvironment [2]. Thus, harnessing macrophages to combat tumors is of longstanding interest [3]. Previous clinical trials demonstrated the feasibility and safety of infusing high doses of autologous monocyte-derived macrophages but failed to demonstrate notable antitumor efficacy [4], suggesting that macrophages require additional signals to direct their activity towards tumors [3]. Insertion of a CAR on macrophages could allow them to selectively recognize and phagocytose antigen overexpressing cancer cells. A few studies have been introducing CARs into macrophages to generate CAR-Macrophages (CAR-M) since 2018, with the objective of directing macrophages’ antitumor function toward tumor cells [3,5,6,7]. To date, preclinical studies on CAR-M have shown promising antitumor activity, and one Phase I clinical trial is ongoing, which uses autologous CAR-M targeting HER2-overexpressing solid tumors [8].

Similar to CAR-T, the core components of CAR-M contain an extracellular domain that provides specific recognition by a single-chain variable fragment (scFv), a hinge domain, a transmembrane domain, and an intracellular domain that presents dedicated downstream signaling. The choice of intracellular domain to direct macrophage antitumor activities is a very important step when developing CAR-M therapies [9]. Several cytoplasmic domains have been explored as components of the CAR-M structure, such as the common γ subunit of Fc receptors (FcRγ), multiple EGF-like-domains protein 10 (Megf10), tyrosine-protein kinase Mer (MerTK), Toll-like receptor 2, and CD3ζ. However, almost all the screening work of CAR-M structures were performed on macrophage cell lines, e.g., J774A.1 and RAW264.7, instead of primary macrophages [10]. Since it is unclear to what extent the macrophage cell lines can mimic primary macrophages, whether or not such a connection can be realized remains unknown. In addition, there are discrepancies between two studies using different macrophage cell lines with CAR bearing the same cytosolic domain [3,7]. Therefore, the careful functional evaluation of CARs with different intracellular domains using primary macrophages is necessary when generating CAR-M architectures.

Besides the genetic modification of the CAR itself, the rational combination of CAR-M with other complementary immunotherapies has the potential to significantly improve the efficacy. CAR-M has potential advantages in homing and infiltrating to solid tumors, while CAR-T has limited infiltrating ability into the dense extracellular matrix of tumor. In addition, the macrophage has the potential to promote T cell activation [11]. Therefore, it is worth investigating whether CAR-M and CAR-T have a synergistic effect against tumor cells. Considering the very limited number of tumor-specific antigens clinically, the combination of CAR-M and CAR-T with CAR targeting the same tumor antigen needs to be explored.

Here, we compared three common engulfment receptor intracellular domains as components of CAR-M on the primary macrophage for their phagocytosis and killing ability. The synergistic effect of CAR-M and CAR-T against tumor cells was investigated, and the preliminary mechanism of action underlying the synergy was clarified.

## 2. Materials and Methods

### 2.1. Cell Culture

Raji cells and K562 cells were obtained from the American Type Culture Collection (ATCC, Manassas, VA, USA). Sp2/0 cells were kindly provided by the Chinese Academy of Sciences Cell Bank/Stem Cell Bank. Cancer cells were cultured in RPMI 1640 medium supplemented with 10% fetal bovine serum (Gibco, Carlsbad, CA, USA), 100 μg/mL streptomycin, and 100 U/mL penicillin (Hyclone, Logan, UT, USA). All the cells were incubated at 37 °C with 5% CO_2_.

### 2.2. Flow Cytometry

For cell-surface staining, cells were incubated with antibodies at 4 °C for 30 min. Subsequent staining was performed with PBS as the staining and wash buffer. TruStain FcX was always used for the FACS staining of the macrophages. In most assays, cells were stained with 7AAD (BioLegend, San Diego, CA, USA) to exclude dead cells.

Anti-mouse FMC63 scFv monoclonal antibody Alexa Fluor 647 (Cat# 300402) was obtained from BioSwan. TruStain FcX™ (anti-mouse CD16/32) antibody (Cat# 101320), Anti-mouse F4/80 antibody PE (Cat# 123110), anti-mouse CD11b antibody APC (Cat# 101212), anti-mouse CD80 antibody PE (Cat# 104708), anti-mouse CD86 antibody APC (Cat# 105012), anti-mouse CD206 antibody BV421 (Cat# 141717), anti-mouse CD44 antibody APC (Cat# 103012), anti-mouse CD62L antibody PerCP (Cat# 104430), anti-mouse CD69 antibody PE (Cat# 104508), anti-mouse TIGIT antibody APC (Cat# 156106), anti-mouse PD-1 antibody PE (Cat# 135206), anti-mouse LAG-3 antibody PerCP/Cyanine5.5 (Cat# 125211), anti-mouse TIM-3 antibody APC (Cat# 134008) and corresponding isotype controls were obtained from BioLegend. Flow cytometry data were acquired on a CytoFLEX LX Flow Cytometer (Beckman Coulter, Pasadena, CA, USA) and analyzed with FlowJo X10 (BD Biosciences, Franklin Lakes, NJ, USA).

### 2.3. Plasmid Construction and Lentivirus Production

**Figure 1:**
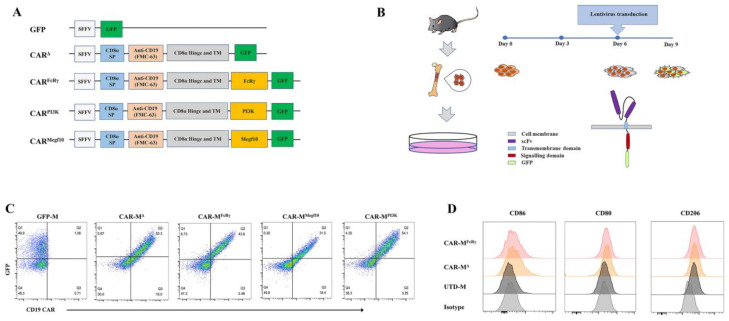
Preparation of CAR-Ms and their CAR expression. (**A**) Schematic representation of lentiviral vectors expressing CAR or GFP control. SP, signal peptide; TM, transmembrane domain. (**B**) Schematic of CAR-M production using lentivirus. (**C**) Flow cytometric analysis of GFP and CAR expression in bone marrow derived macrophages (BMDM). Data are representative of three independent experiments. (**D**) Flow cytometric analysis of macrophage polarization M1 and M2 surface markers (CD86, CD80, CD206). UTD-M, untransduced macrophage. Data are representative of three independent experiments.

The pHR CD19-FcRγ CAR vector was a gift from Ron Vale (Addgene plasmid # 113014) [3]. All other lentiviral vectors were constructed or truncated based on the pHR CD19-FcRγ CAR vector following the Gibson assembly protocol. Synthetic CARs (Figure 1A) contained the human CD8α signal peptide followed by the scFv of anti-CD19 (clone FMC63) linked in-frame to the hinge and transmembrane domain of the human CD8α molecule, and intracellular signaling domains of FcRγ (aa 45–86 of Mouse FcRγ, Uniprot P20491), Megf10 (aa 879–1147 of Mouse Megf10, Uniprot Q6DIB5), the cytoplasmic domain that recruits the p85 subunit of PI3K (aa 500–534 of Mouse CD19, Uniprot P25918). The detailed sequence information is shown in Table S1. For the lentiviral package, the lentiviral plasmids were cotransfected into HEK293T cells with the packaging plasmids psPAX2 and pMD2.G at a ratio of 5:3:2. Lentivirus was harvested 48 h after transfection.

### 2.4. CAR-M Cells Production

Bone marrow derived macrophages (BMDM) were produced as previously described [12], except that L-929 conditioned media was replaced with purified 20 ng/mL M-CSF (Biolegend). The BMDM were infected with lentivirus supernatant for 24 h after 6 days of differentiation. The CAR-Ms were harvested on day 9 for the further tests (Figure 1B).

### 2.5. Murine CAR-T Cells Production

Retroviral supernatants used for transduction and murine CAR-T cells were prepared as previously described [13]. Briefly, retrovirus for mouse T cell transduction was generated using packaging vector encoding Eco envelope protein. Mouse T cells were isolated using the Mojosort T cell isolation kit (BioLegend) from splenocytes obtained from C57BL/6J mice. T cells were then stimulated for 48 h, followed by transduction with retroviral supernatant using retronectin-coated plates. T cells were expanded in complete medium with IL-7 and IL-15, changing the medium every 2 days. On day 5, CAR-T cells were collected and used for subsequent assays.

### 2.6. Phagocytosis Assay

The microscopy-based phagocytosis assay was performed as previously described with some modification [3,14]. Briefly, CAR-Ms normalized for transduction efficiency were plated at 5 × 10^4^ cells/well in 24-well tissue culture plates one day before the phagocytosis assay. Raji cells were labeled with CellTrace Violet (Thermo Fisher, Waltham, MA, USA) according to the manufacturer’s instruction. After incubating CAR-Ms in serum-free IMDM medium for 2 h, 2 × 10^5^ CellTrace Violet-labeled Raji cells were added to preseeded CAR-Ms. After 2 h coculture, Raji cells were washed 3 times with ice-cold PBS to remove the unengulfed ones. All samples were imaged with an inverted fluorescence microscope (Olympus IX83). Phagocytosis was determined by counting Raji cells engulfed by CAR-Ms using ImageJ. Phagocytosis efficiency of CAR-Ms was calculated as the number of Raji cells engulfed by per 100 CAR-M.

The FACS-based phagocytosis assay was performed as previously described [14]. Briefly, CAR-Ms were incubated with CellTrace Violet-labeled Raji cells as detailed for the microscopy-based phagocytosis assay, except that the plates used were nontreated. Once the phagocytosis period was completed, all cells in the well were collected in the presence of Accutase (Biolegend) and analyzed by FACS. Phagocytosis efficiency was determined as the percentage of CAR-M containing blue fluorescence.

### 2.7. Cytotoxicity Assays

FACS counting-based killing assay was performed as previously described with some modification [3]. Briefly, CAR-Ms or control were plated in 48-well tissue culture plates one day before the cytotoxicity assay, then CellTrace Violet-labeled target tumor cells were added to the plate. After 24 h or 48 h coculture, the remaining number of target tumor cells remaining was analyzed by FACS as follows: 10,000 CountBright^TM^ absolute counting beads (Thermo Fisher) were added to the well immediately prior to reading and the cell-counting bead mixture was harvested by pipetting up and down. Percentage cytotoxicity was calculated as: % cytotoxicity = [(1 − (the remaining number of target cells from treated groups/the number of target cells alone)] × 100.

Luciferase-base killing assay was performed as previously described [5]. Briefly, target tumor cells expressing luciferase were cultured with effector cells for 48 h in a white-walled 96-well plate. D-luciferin (150 μg/mL) was added 10 min prior to the bioluminescence reading in a SPECTROstar Omega microplate reader (BMG Labtech, Ortenberg, Baden-Württemberg, Germany). Percent specific lysis was calculated on the basis of luciferase signal (total flux) relative to tumor alone, using the following formula: % specific lysis = [(Sample signal − Tumor alone signal)/(Background signal − Tumor alone signal)] × 100.

### 2.8. Cytokine/Chemokine Analysis

Cytokine/chemokine multiplex analysis was carried out using Luminex xMAP technology [15]. The analysis was performed on supernatants derived from cultures given the indicated treatments. Supernatants of Raji coculture system with GFP-macrophage (M), CAR-M^FcRγ^, T, CAR-T, or the combination of CAR-M^FcRγ^ and CAR-T (CAR-M^FcRγ^ + CAR-T) for 48 h were analyzed using magnetic MILLIPLEX MAP antibody-conjugated beads (Merck-Millipore) according to the manufacturer’s instructions on a Luminex MAGPIX instrument with the xPONENT 4.2 software (Luminex, Austin, TX, USA). A panel of 18 murine cytokine/chemokine was measured, including GM-CSF, IFN-γ, IL-1α, IL-1β, IL-2, IL-4, IL-5, IL-6, IL-7, IL-10, IL-12(p70), IL-13, CXCL5, IL-17A, CXCL1, MCP-1, MIP-2, TNF-α.

### 2.9. Statistical Analysis

Data are shown as mean ± SD, with each condition in at least triplicate. Statistical significance was calculated using the two-tailed Student t-test or one-way ANOVA analysis. 

## 3. Results and Discussion

### 3.1. Generation of Three Different CAR-Ms and Comparison of their Phagocytic and Killing Functions

The choice of intracellular domain is of great importance when designing CAR-M. The validation of potent signaling domain on primary macrophage is an essential step towards a possible therapeutic option. Therefore, we sought to screen the best intracellular domain on primary murine macrophages, BMDM.

To this end, we first optimized the culture conditions to obtain a high number of BMDM. The concentrations of FBS and murine M-CSF were used as main determining factors for optimization. The results showed that RPMI-1640 medium with 20% FBS and 20 ng/mL M-CSF was the best medium condition, in which the largest number of BMDM was harvested (Figure S1A,B). The purity of BMDM was around 99% as determined by F4/80 and CD11b expression (Figure S1C). To identify the best cytoplasmic domain capable of promoting phagocytosis and cytotoxicity, three effective phagocytic receptor intracellular domains were selected as components of CARs, which are CAR^FcRγ^, CAR^Megf10^, and the cytoplasmic domain that recruits the p85 subunit of PI3K (CAR^PI3K^). All the three selected intracellular domains have been previously validated to promote phagocytosis of antigen-ligated beads on CAR-macrophage cell lines [3]. CD3ζ intracellular domain was used on CAR-M in the clinical trial. We did not include CD3ζ in our comparation list, since CD3ζ and FcRγ have been demonstrated to be functionally similar on human primary macrophage [5], and CD3ζ is not naturally present in macrophage. The three selected intracellular domains were fused with a CD8α signal peptide, FMC63 scFv specifically binding to human CD19, CD8α hinge and transmembrane domain and GFP tag. Additionally, a construct without any intracellular domain (CAR^Δ^) and a construct with only a GFP tag were generated as controls (Figure 1A). All the CARs can be highly expressed on BMDM by means of the established CAR-M production platform (Figure 1B,C). Then, a CAR positive rate of 30% on CAR-M was set as a cutoff for further activity test. In addition, macrophage polarization M1 and M2 surface markers (CD86, CD80, CD206) were analyzed for macrophages with or without lentivirus transduction. The data showed that the BMDM differentiated and matured using M-CSF expressed CD206, which was consistent with the report that M-CSF stimulation induces an M2-like phenotype in macrophages [16]. Compared with untransduced macrophage (UTD-M), CAR-M transduced by lentivirus possessed more M1 features with more CD86 and CD80 expression (Figure 1D; Figure S1D). It implies that the lentiviral vector used to transduce macrophages with CAR probably induce CAR-M M1 phenotype polarization.

**Figure 2:**
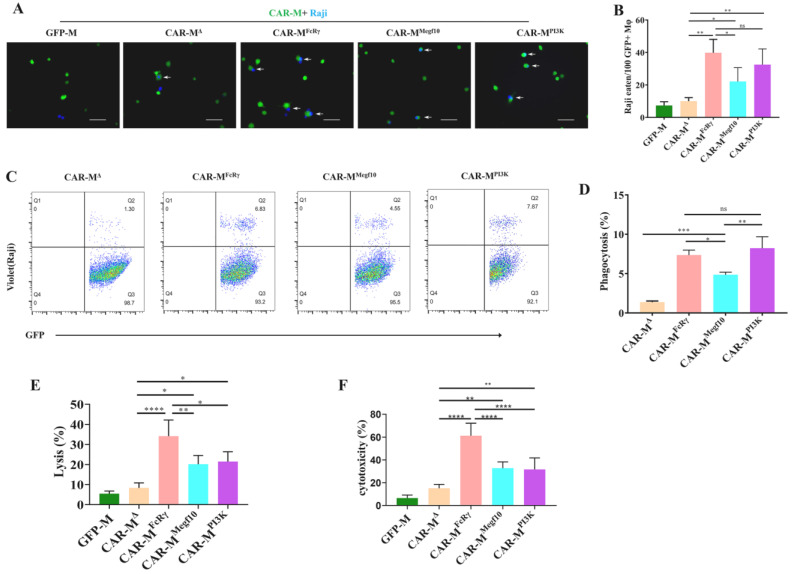
The comparation of phagocytic and killing capacity of different CAR-Ms. (**A**) Phagocytosis of Raji by different CAR-Ms was assessed using fluorescence microscopy. Scale bars, 50 μm; CAR-M, green; targets, blue. Photographs are representative of six. (**B**) Graph depicts pooled data from phagocytosis photographs of panel (**A**). The results are presented as the number of Raji eaten by 100 GFP+ macrophage (Mφ). All data are means ± SD, *n* = 6. (**C**) Phagocytosis of Raji by different CAR-Ms was assessed using flow cytometry. Plots are representative of three. (**D**) Graph depicts pooled data from panel (**C**). Quantification of phagocytosis of Raji cells by flow cytometric analysis (*n* = 3). (**E**) Luciferase-based killing assay of Raji cell by GFP-M control or CAR-Ms at 48 h. Data represent the mean ± SD of *n* = 3–4 biological replicates. (**F**) FACS counting-based killing assay of Raji by GFP-M control or CAR-Ms at 48 h. Data represent the mean ± SD of *n* = 3 biological replicates. Statistical significance was calculated using one-way ANOVA (* *p* ≤ 0.05, ** *p* ≤ 0.01, *** *p* ≤ 0.001, **** *p* ≤ 0.0001).

The phagocytic and killing capacity of CAR-Ms to antigen-positive tumor cells are the most direct and convincing indicators for comparing different CARs. Microscopy- and FACS-based phagocytosis assessment of CAR-Ms towards cancerous Raji cells that express high levels of CD19 was performed. CAR-M cells normalized for transduction efficiency were used in these assays. All three CAR-Ms were able to trigger engulfment of Raji cells. CAR-M^FcRγ^ and CAR-M^PI3K^ could engulf more target cells than CAR-M^Megf10^, while CAR-M^FcRγ^ and CAR-M^PI3K^ showed comparable phagocytic capacity (Figure 2A–D). Cytotoxicity of CAR-Ms against Raji was performed using the luciferase-based and FACS counting-based killing assay. The three CAR-Ms also showed potent cytotoxicity against Raji, and CAR-M^FcRγ^ was demonstrated to be the most effective (Figure 2E,F). CAR-M^FcRγ^ performed better than CAR-M^PI3K^ in terms of killing capacity, though they had comparable phagocytic capacity. CAR-M can initiate both whole cell eating and trogocytosis leading to cancer cell elimination [3], and our killing assays do not distinguish between whole cell engulfment or death following trogocytosis.

**Figure 3:**
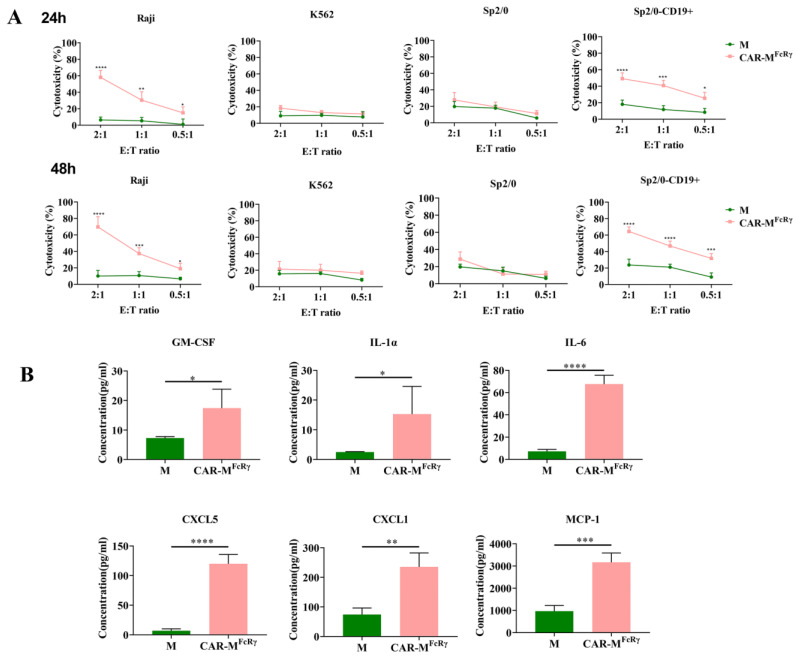
The cytotoxicity of CAR-M^FcRγ^ against antigen-positive and antigen-negative cells and cytokine/chemokine analysis. (**A**) FACS counting-based killing assay of Raji, K562, Sp/20, Sp2/0-CD19+ cells by GFP-M (M) control or CAR-M^FcRγ^ at 24 or 48 h at different E:T ratios (2, 1 or 0.5). Data represent the mean ± SD of *n* = 3 biological replicates. (**B**) Cytokine/chemokine released by M or CAR-M^FcRγ^ after coculture with Raji for 48 h at an E:T ratio of 2. Data are represented as the mean ± SD of *n* = 3 biological replicates. Statistical significance was calculated using Student’s *t*-test (* *p* ≤ 0.05, ** *p* ≤ 0.01, *** *p* ≤ 0.001, **** *p* ≤ 0.0001).

After investigation of CAR constructs with different intracellular domains, we found that CAR-M^FcRγ^ has the most potent phagocytic and killing capacity to Raji cells among the tested three CAR-Ms. To validate that CAR-M^FcRγ^ triggers antigen-specific cellular killing effects, Raji, human CD19-negative tumor cell K562, murine cancer cell Sp2/0, and human CD19-overexpressed Sp2/0 (Sp2/0-CD19+) cells were used as target cells for cytotoxicity assessment. The killing assay was performed at different time points (24 h and 48 h) and different E:T ratios (2, 1 or 0.5). The data indicate that CAR-M^FcRγ^ exerted potent specific cytotoxicity function in a dose- and time-dependent manner against CD19-positive cells compared with the GFP-M (M) control (Figure 3A). Additionally, a panel of 18 cytokine/chemokine (GM-CSF, IFN-γ, IL-1α, IL-1β, IL-2, IL-4, IL-5, IL-6, IL-7, IL-10, IL-12(p70), IL-13, CXCL5, IL-17A, CXCL1, MCP-1, MIP-2, TNF-α) analysis was performed for the supernatant of M control or CAR-M^FcRγ^ and Raji coculture system. The levels of GM-CSF, IL-1α, IL-6, CXCL5, CXCL1 and MCP-1 in the CAR-M^FcRγ^ group were significantly higher than the M control in various degrees (Figure 3B), while the other cytokines/chemokines did not display significant difference (data not shown). These data indicate that CAR-M^FcRγ^ were activated by Raji cells and induced an inflammatory response. The significantly increased proinflammatory cytokines and chemokines could be beneficial for antitumor immunity. Moreover, Klichinsky et al. [5] have shown that human CAR-M with a CD3ζ intracellular domain generated by an Ad5-F35 vector can reprogram the tumor microenvironment (TME) by releasing proinflammatory cytokines that can activate innate immune cells and achieve a remarkable antitumor effect in vivo.

### 3.2. CAR-M and CAR-T Cells Demonstrated Synergistic Cytotoxicity

Despite the effective tumoricidal activity of CAR-M, given the complexity and difficulty of treating cancer, rationally combining CAR-M therapy with other complementary immunotherapy is a potential strategy in combating cancer [17]. The use of combination therapy to simultaneously target different mechanisms of action has proven to be a viable approach to treat cancer [18]. CAR-M has potential advantages in homing and infiltrating to solid tumor and can release proinflammatory cytokines to improve TME. Despite rapid advances in CAR-T immunotherapy, clinical responses in solid tumors have been limited. The major obstacles of CAR-T cell therapy to treat solid tumors include limited infiltration into the dense extracellular matrix of tumor and exhaustion in immunosuppressive TME [19]. It is generally accepted that CAR-M could leverage the natural tumor-homing ability of myeloid cells to enter solid tumors, which is superior to CAR-T [20]. Though the comparison of the homing capacity between CAR-M and CAR-T has not been confirmed experimentally, it is foreseen in future studies. Moreover, the macrophage has the potential to promote T cell activation. Therefore, it is likely that CAR-M and CAR-T can complement each other and significantly augment tumor responses. Considering the very limited number of tumor-specific antigens clinically, the combination of CAR-M and CAR-T with CAR targeting the same tumor antigen is realistic. On the other hand, trogocytosis occurs during CAR-T killing target tumor cells, in which the target antigen is transferred to CAR-T cells, thereby abating T cell activity by promoting fratricide CAR-T-cell killing [21]. It is uncertain whether CAR-M would attack antigen-positive CAR-T cells and lessen the overall tumoricidal activity when CAR-M and CAR-T were combined. Therefore, the real experiment needs to be performed to investigate whether the combination of CAR-T and CAR-M targeting the same tumor antigen have a synergistic, additive, or antagonistic effect against tumors.

**Figure 4:**
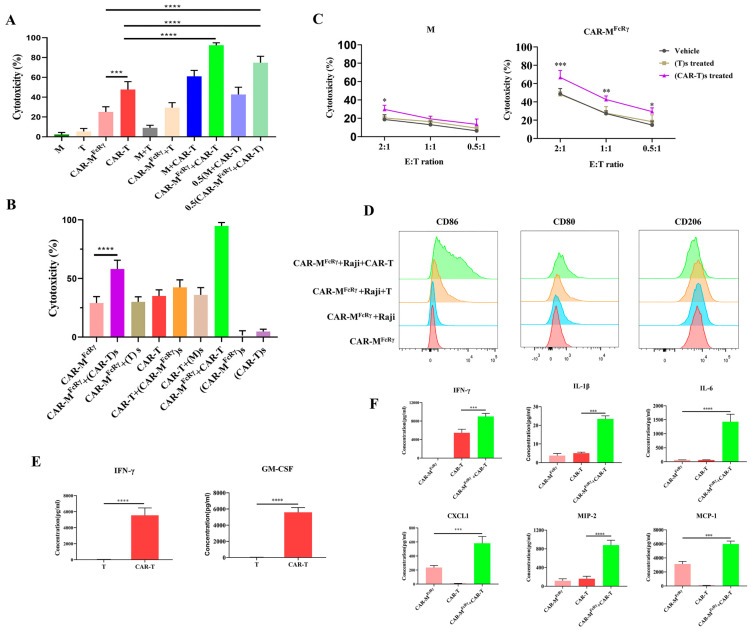
CAR-T and CAR-M demonstrated synergistic cytotoxicity against target cells. (**A**) Cytotoxicity assay against Raji by different immune effector cells alone or different combination regimens after 48 h coculture at an E:T ratio of 1 (M, T, CAR-M^FcRγ^, CAR-T, 0.5 (CAR-M^FcRγ^ + CAR-T), 0.5 (CAR-M^FcRγ^ + CAR-T)) or 2 (M + T, CAR-M^FcRγ^ + T, M + CAR-T, CAR-M^FcRγ^ + CAR-T). M, GFP-M; T, untransduced T cells. (**B**) Cytotoxicity assay against Raji by different immune effector cells with normal media or media supplemented with 50% supernatant of immune effector cells and Raji coculture system. The supernatants of T, CAR-T, M or CAR-M^FcRγ^ with Raji coculture system after 48 h at an E:T ratio of 2 were referred to as (T)s, (CAR-T)s, (M)s and (CAR-M^FcRγ^)s, respectively. (**C**) M or CAR-M^FcRγ^ was treated by (T)s or (CAR-T)s for 24 h prior to cytotoxicity assay against Raji. Assays were performed after 48 h coculture at different E:T ratios (2, 1 or 0.5). (**D**) Flow cytometric analysis of macrophage polarization M1 and M2 surface markers (CD86, CD80, CD206) of CAR-M^FcRγ^ alone or cocultured with Raji, Raji + T, Raji + CAR-T for 48 h at an E:T (T or M effector cell: Raji) ratio of 2. (**E**) IFN-γ and GM-CSF released by T or CAR-T cells after coculture with Raji for 48 h at an E:T ratio of 2. (**F**) Cytokine/chemokine released by CAR-T, CAR-M^FcRγ^, or the combination of CAR-T and CAR-M^FcRγ^ (CAR-M^FcRγ^ + CAR-T) after coculture with Raji for 48 h at an E:T ratio (T or M effector cell: Raji) of 2. Data are represented as the mean ± SD of *n* = 3 biological replicates. Statistical significance was calculated using two-tailed Student t-test or one-way ANOVA analysis (* *p* ≤ 0.05, ** *p* ≤ 0.01, *** *p* ≤ 0.001, **** *p* ≤ 0.0001).

Murine CD19 CAR-Ts with a classic second-generation CAR structure (Figure S2) were prepared for the combinational study with CAR-M^FcRγ^ against Raji cells. Untransduced T and GFP-M (M) cells were employed as controls. Immune effector cells (T, CAR-T, M and CAR-M^FcRγ^) were added either alone or in combination with the counterpart effector cells to kill Raji cells. Of interest, CAR-T showed more potent cytotoxicity than CAR-M^FcRγ^ at the same E:T ratio (E:T = 1). CAR-T + CAR-M^FcRγ^ combination significantly augmented the cytotoxicity compared with CAR-T or CAR-M^FcRγ^ alone, in which this increase exceeded simple additive effects. Of note, the 0.5 (CAR-M^FcRγ^ + CAR-T) group, which combined half the dose of CAR-T alone and CAR-M^FcRγ^ alone group (0.5 + 0.5), demonstrated significantly greater killing ability than CAR-T or CAR-M^FcRγ^ alone as well (Figure 4A). This greater-than-expected tumor killing indicates that CAR-M^FcRγ^ and CAR-T have synergistic effect against Raji cells. In addition to CAR-M^FcRγ^, the combination of CAR-T with other CAR-Ms (namely CAR-M^Megf10^ and CAR-M^PI3K^) were assessed as well. CAR-T also synergized with other CAR-Ms in terms of killing Raji using the luciferase-based cytotoxicity assay (Figure S3A). Moreover, synergistic effect occurred similarly after the coculture with the murine cancer cell, Sp2/0-CD19+ (Figure S3B). Taken together, these data demonstrate for the first time that CAR-M synergize with CAR-T therapy against tumor cells. The combination approach offers a novel strategy for treating cancer.

### 3.3. The Mechanism Underlying the Synergistic Effect of CAR-M and CAR-T

Clarifying the mechanism underlying the synergistic effect of CAR-M and CAR-T is beneficial for the further development of the combination therapy. We hypothesized that the cytokines released by CAR-T or CAR-M may be involved in the synergy. To explore the possible mechanism, the supernatants of T or CAR-T with the Raji cells coculture system after 48 h of killing, hereafter referred to as (T)s or (CAR-T)s, were collected to supplement the coculture system of M or CAR-M^FcRγ^ killing Raji. Similarly, the supernatants of M or CAR-M^FcRγ^ with Raji cells coculture system after 48 h of killing, hereafter referred to as (M)s or (CAR-M^FcRγ^)s, were collected to supplement the coculture system of T or CAR-T killing Raji. The cytotoxicity of (CAR-T)s and (CAR-M^FcRγ^)s alone were also performed to exclude their possible direct killing ability against Raji. After killing for 48 h, the results showed that CAR-M^FcRγ^ exposing to (CAR-T)s achieved more potent activity than CAR-M^FcRγ^ alone, while CAR-T exposing to (CAR-M^FcRγ^)s did not improve the killing ability. Moreover, (CAR-T)s or (CAR-M^FcRγ^)s alone showed no potent direct cytotoxicity (Figure 4B). To further validate the effect of (CAR-T)s on CAR-M^FcRγ^, M or CAR-M^FcRγ^ were exposed to (T)s or (CAR-T)s for 24 h followed by the removal of cell culture supernatants. The cytotoxicity assay was performed at different E:T ratios. CAR-M^FcRγ^ exposed to (CAR-T)s demonstrated more potent cytotoxicity than untreated and (T)s treated CAR-M^FcRγ^. M treated by (CAR-T)s also showed slightly enhanced cytotoxicity, but to a less extent than CAR-M (Figure 4C). This also indicates that (CAR-T)s pretreatment enhanced the killing ability of M, while a CAR can amplify this effect. The similar phenomenon was also observed using the murine cancer cell Sp2/0-CD19+ as the target cell (Figure S3C). Taken together, CAR-T-derived cytokines can enhance the killing activity of CAR-M, which contributes to the synergistic effect of CAR-M and CAR-T.

Additionally, the polarization phenotype of CAR-M from killing assay in combination with CAR-T was analyzed. The expression of CD86 and CD80 on CAR-M^FcRγ^ was significantly upregulated after the coculture with CAR-T and Raji for 48 h, while CD206 was downregulated (Figure 4D; Figure S4A). However, Raji cells alone could not significantly trigger the upregulation of CD80 or CD86 on CAR-M^FcRγ^. The similar trend was observed when CAR-M were treated with (CAR-T)s for 24 h (Figure S4B,C). This suggests that CAR-M were polarized to a more M1 phenotype by CAR-T cell-derived cytokines. Inflammatory stimuli (e.g., IFN-γ, GM-CSF) have been shown to polarize macrophages toward a M1 phenotype, that favors the elimination of tumor cells [22]. We also observed a high level of IFN-γ and GM-CSF secreted by CAR-T during killing Raji cells (Figure 4E). These data reveal that the inflammatory cytokines (including but not limited to IFN-γ and GM-CSF) released by CAR-T during killing tumor cells probably polarize CAR-M toward a more M1 phenotype, which augments the cytotoxicity of CAR-M.

Given that inflammatory stimuli augment the ability of macrophages to phagocytose and destroy cancer cells, we sought to take advantage of the principle to design more potent CAR-M. A very recent mechanism study demonstrated that CD18 of macrophage is crucial for the increased phagocytosis and antitumor activity in inflammatory settings [22]. Therefore, we sought to improve the killing capacity of CAR^FcRγ^ by assembling the intracellular domain of CD18 with FcRγ in a tandem array to construct CAR^FcRγ-CD18^ (Figure S5A). The cytotoxicity of CAR-M^FcRγ-CD18^ and CAR-M^FcRγ^ were compared in parallel. However, an additional CD18 intracellular domain failed to augment the cytotoxicity (Figure S5B). The transmembrane domain and/or extracellular domain of CD18 may be necessary for the improved phagocytic capacity of inflammatory macrophages. Further investigation is required to determine the precise cause of this unexpected outcome.

CD86 and CD80 on the macrophage are the ligands of costimulatory molecule CD28 on CAR-T cells, which contribute a positive costimulatory signal during T cell priming and activation [23]. The upregulated CD86 and CD80 on CAR-M induced by CAR-T secreted cytokines may amplify the magnitude of CAR-driven signals and mediate a set of signaling events that influence CAR-T cell killing ability in turn. Furthermore, the cytokine/chemokine analysis showed that IFN-γ, IL-1β, CXCL1, MIP-2, IL-6 and MCP-1 were significantly increased when CAR-T and CAR-M^FcRγ^ were combined (Figure 4F), indicating that the interaction of CAR-M and CAR-T may encourage mutual activation and can form an inflammatory TME. On the other hand, attention should be paid to the possible cytokine storm resulted from the combination of CAR-M and CAR-T in the clinic in the future. It has been shown that the main cytokine responsible for Cytokine Release Syndrome (CRS) in recipients of CAR-T is IL-6 produced by recipient macrophages cells [24]. Maybe the only way to evaluate a potential beneficial or detrimental effect of CAR-M cytokine secretion will be with in vivo testing.

**Figure 5:**
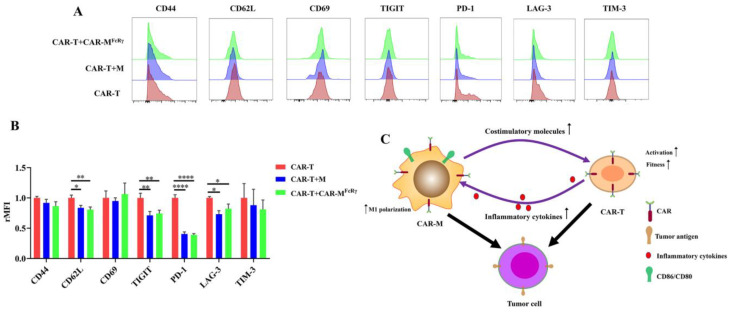
CAR-T phenotype change after coculture with Raji alone, or in the presence of GFP-M (M) or CAR-M. (**A**) Flow cytometric analysis of CD44 CD62L CD69, PD-1, LAG-3, TIM-3, TIGIT of CAR-T cells cocultured with Raji alone, or together with M or CAR-M^FcRγ^ for 48 h at an E:T (T or M effector cell: Raji) ratio of 2. Data are representative of three independent experiments. (**B**) Quantification of surface markers CD44, CD62L, CD69, PD-1, LAG-3, TIM-3, TIGIT expression in CAR-T cells from panel (**A**). rMFI, relative mean fluorescence intensity. (**C**) Schematic representation of possible mechanisms for the synergistic effect of CAR-T and CAR-M^FcRγ^ against target cells. Data are represented as the mean ± SD of *n* = 3 biological replicates. Statistical significance was calculated using one-way ANOVA (* *p* ≤ 0.05, ** *p* ≤ 0.01, **** *p* ≤ 0.0001).

To further explore the impact of CAR-M on CAR-T cells in detail, activation/memory markers (CD44, CD62L, and CD69) and exhaustion markers (PD-1, TIM-3, TIGIT, and LAG-3) on CAR-T cells were analyzed after exposure to Raji alone or in the presence of CAR-M^FcRγ^ or M. The expression of CD62L of CAR-T in the presence of CAR-M^FcRγ^ or M significantly decreased (Figure 5A,B). CD62L has be used as a marker of T cell activation [25]. It suggests that CAR-M or M may facilitate the activation of CAR-T cells. In addition, the presence of CAR-M^FcRγ^ or M decreased the expression of exhaustion markers PD-1, TIGIT, and LAG-3 of CAR-T cells, which indicates that the fitness of CAR-T was improved by CAR-M^FcRγ^ after exposure to Raji in vitro (Figure 5A,B). Taken together, the presence of CAR-M^FcRγ^ is beneficial for CAR-T to kill tumor cells, through promoting the level of activation and fitness of CAR-T after exposure to target tumor cells. It could be due to the upregulated CD28 ligands, CD86 and CD80, on the macrophage [23]. The detailed mechanism underlying this phenomenon awaits further study.

Overall, the synergistic effect of CAR-M and CAR-T against tumor cells probably depend on a feedback loop triggered by the activation of CAR-T. The inflammatory factors secreted by CAR-T increase the expression of costimulatory ligands (CD86 and CD80) on CAR-M and augment the cytotoxicity of CAR-M by inducing macrophage to M1 polarization. The upregulated costimulatory ligands may promote the fitness and activation of CAR-T cells in turn, ultimately achieving significantly enhanced cytotoxicity (Figure 5C). Considering the mechanism underlying the synergistic effect of CAR-T and CAR-M in our study, the use of CAR-M and other second generation CAR-T cells (e.g., 41BB-CD3ζ CAR-T) could show the similar synergistic effect. The presence of the costimulatory domain of CAR-T probably play an important role in the synergistic effect, since the costimulatory domain contributes to CAR-T cells persistence and the sustained high level of inflammatory factors released by CAR-T during the killing of tumor cells [26].

In this study, we revealed that CAR-M and CAR-T cells synergistically kill tumor cells in vitro and provided rationale for the combination of CAR-M with CAR-T to treat cancer. Despite the achievements made, as the tumor microenvironment in vivo is highly intricate, further studies are required to evaluate the synergistic effect of CAR-M and CAR-T in vivo. Moreover, the detailed analysis of the tumor immune microenvironment after the CAR-T and CAR-M combinational treatment in a syngeneic immunocompetent animal model can deepen our understanding to adoptive immune-cell therapy, which may aid in developing more effective therapies. Additionally, the combination of CAR-M and CAR-T targeting different tumor antigens probably demonstrate a more profound synergistic effect, in which tumor cells with either of two different antigens could be directly killed and the fratricidal killing due to CAR-T cell trogocytosis may be limited [21]. The potential drawback of the combination of CAR-M and CAR-T targeting different tumor antigens in the clinic could be the safety issue, given the limited number of tumor specific antigens.

Although CAR-M therapy has shown its effective antitumor ability in animal experiments, it also has many shortcomings to be overcome. Therefore, future effort should be given to maximize the effectiveness and safety of CAR-M in clinical treatment. First, the previously reported achievements, including our study, are all based on the first generation of CAR-M, in which the intracellular signaling is mediated by a single effector domain [3,5,6,7]. Therefore, CAR structure can be optimized by incorporating tandem activation domains or proinflammatory cytokines (e.g., IFN-γ, GM-CSF) to enhance its effectiveness [27]. Multiantigen logic gates or drug-sensitive modules can also be designed to engineer CAR-M to improve its safety. Additionally, considering the laborious and costly process of ex vivo autologous CAR-M cell production, in vivo programming of the macrophage into CAR-M using nonviral delivery of CAR-encoding DNA or mRNA is a promising strategy [28,29]. Lastly, combining CAR-M therapy with other therapeutics will be of great benefit to patients, especially those with high tumor burden. In addition to CAR-T therapy in this study, chemotherapies, CD47/SIRPa antibodies, T cell checkpoint inhibitors, or radiation therapy can also be carefully evaluated for the possible synergy with CAR-M, aiming to significantly improve the overall therapeutic efficacy.

## 4. Conclusions

Collectively, we generated three CAR-Ms with different intracellular domains using primary macrophages BMDM and compared their phagocytic and killing capacity towards tumor cells. CAR-M^FcRγ^ showed the most potent phagocytic and killing capacity among the three CAR-Ms. Notably, this work also demonstrated for the first time that CAR-M could synergize with CAR-T cells. The synergistic effect could be ascribed to a feedback loop, in which the inflammatory factors secreted by CAR-T augment the cytotoxicity of CAR-M by inducing macrophage M1 polarization and increase the expression of costimulatory ligands on CAR-M, that may promote the fitness and activation of CAR-T cells in turn.

## Supplementary Materials

The following supporting information can be downloaded at: https://www.mdpi.com/article/10.3390/cells11223692/s1, Figure S1: Bone marrow derived macrophages (BMDM) harvested with different medium and macrophage polarization M1 and M2 surface markers expression in different macrophages after lentiviral transduction; Figure S2: Construction and identification of murine CD19 CAR-T; Figure S3: CAR-T and CAR-M demonstrated synergistic cytotoxicity against murine target cell Sp2/0-CD19+; Figure S4: The polarization phenotype of CAR-M^FcRγ^ from killing assay in combination with CAR-T or treated by the supernatants of CAR-T with Raji cells coculture system; Figure S5: Comparation of cytotoxicity of CAR-M^FcRγ-CD18^ and CAR-M^FcRγ^.
