# Supplementary material for: CAR-Macrophages and CAR-T Cells Synergistically Kill Tumor Cells In Vitro
Source: Cells. 2022 Nov 21;11(22):3692. doi: 10.3390/cells11223692 (PMC9688246; doi:10.3390/cells11223692)
Supplement: Supplementary file 1 [file cells-11-03692-s001.zip › cells-1993792-supplementary.pdf]

cells

The Supplementary Materials for

Table S1. CAR component sequences.

**Signal peptide:**	
**CD8α signal peptide**	MALPVTALLLPLALLLHAARP
**ScFv:**	
CD19 scFv (FMC63)	DIQMTQTTSSLSASLGDRVTISCRASQDISKYLNWYQQKPDGTVKLLI-<br>YHTSRLHSGVPSRFSGSGSGTDYSLTISNLEQEDIATYFCQQGNTLPYTFGGGTKLEITGGG<br>GSGGGGSGGGGSEVKLQESGPGLVAPSQSLSVTCTVSGVSLPDYGVSWIRQP-<br>PRKGLEWLGVIWGSETTYYNSALKSRLTIIKDNSKSQVFLKMNSLQTDDTAIYYCAKHYYY<br>GGSYAMDYWGQGTSVTVSS
**Hinge and transmembrane domain:**	
CD8α hinge and Transmembrane	TTTPAPRPPTPAPTIASQPLSLRPEACRPAAGGAVHTRGLDFACDIYIWAPLAG-<br>TCGVLLLSLVITLYC
**Intracellular signaling domains:**	
FcRγ	RLKIQVRKAAIASREKADAVYTGLNTRSQETYETLKHEKPPQ
PI3K	LYAAPQLHSIQSGPSHEEDADSYENMDKSDDLEPA
Megf10	YRHKQKRKESSMPAVTYTPAMRVINADYTIAETLPHSNGGNANSHYFTNPSYHT-<br>LSQCATSPHVNNRDRMTIAKSKNNQLFVNLKNVNPGKRGTLVDCTGTLPADWKQGGYL<br>NELGAFGLDRSYMGKSLKDLGKNSEYNSSTCSLSSSENPYATIKDPPALLPKS-<br>SECGYVEMK-<br>SPARRDSPYAEINNSTPANRNVYEVEPTVSVVQGVFSNSGHVTQDPYDLPKNSHIPCHYDL<br>LPVRDSSSSPKREDGGGSNSTSSNSTSSSSSSSE
**Linker between CAR and GFP:**	
Linker between CAR and GFP	GSGS
**GFP:**	
GFP	MVSKGEELFTGVVPILVELDGDVNGHKFSVSGEGEGDATYGKLTLKFICTTGKLPVPWPT-<br>LVTTLTYGVQCFSRYPDHMKQHDFFKSAMPEGYVQERTIFFKDDGNYKTRAEVKFEGDTL<br>VNRIELKGIDFKEDGNILGHKLEYNYNSHNVYIMADKQKNGIKVNFKIR-<br>HNIEDGSVQLADHYQQNTPIGDGPVLLPDNHYLSTQSALSKDPNEKRDHMVLLEFVTAA<br>GITLGMDELYK

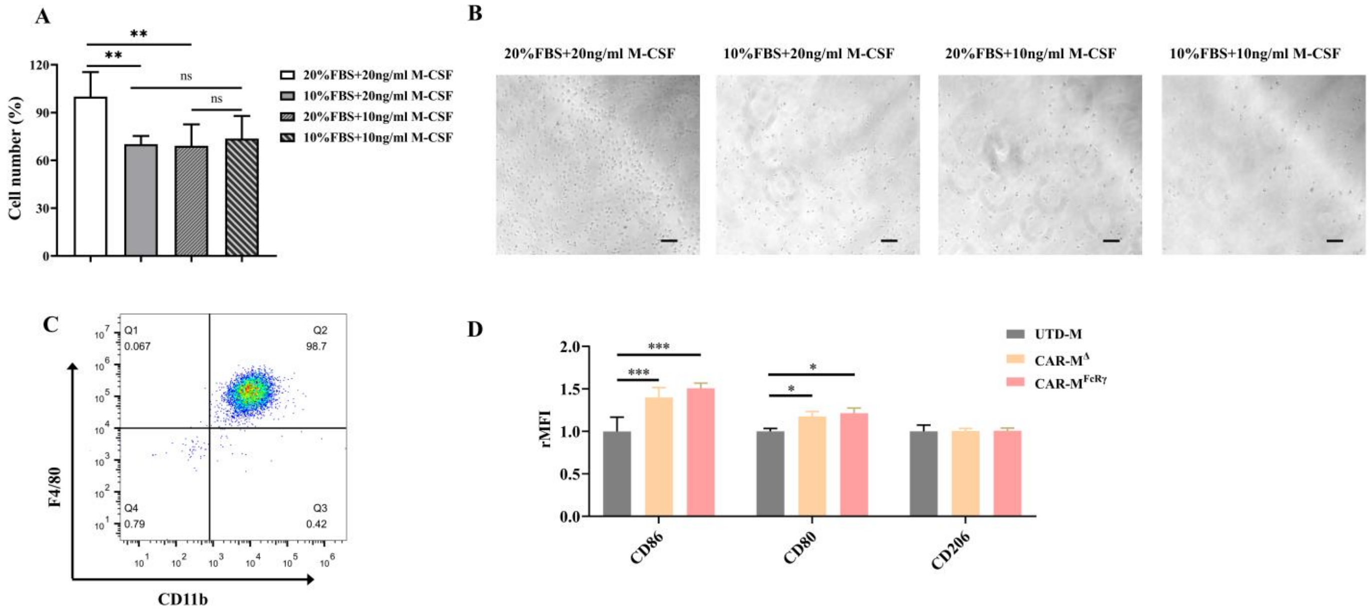

Figure S1. Bone marrow derived macrophages (BMDM) harvested with different medium and macrophage polarization M1 and M2 surface markers expression in different macrophages after lentiviral transduction. (**A**) Cell number of BMDM harvested after culture with different medium for 6 days. (**B**) Images of BMDM cultured with different medium for 6 days. Scale bar = 100 μm. (**C**) Flow cytometry characterization of BMDM using F480 and CD11b. (**D**) Quantification of surface markers (CD86, CD80, CD206) expression in different macrophages after lentiviral transduction from (Figure 1D). Data are represented as the mean ± SD of $n = 3$ biological replicates. UTD-M, untransduced macrophage; rMFI, relative mean fluorescence intensity. Statistical significance was calculated using one-way ANOVA analysis ($*p \leq 0.05$, $**p \leq 0.01$), $***p \leq 0.001$).

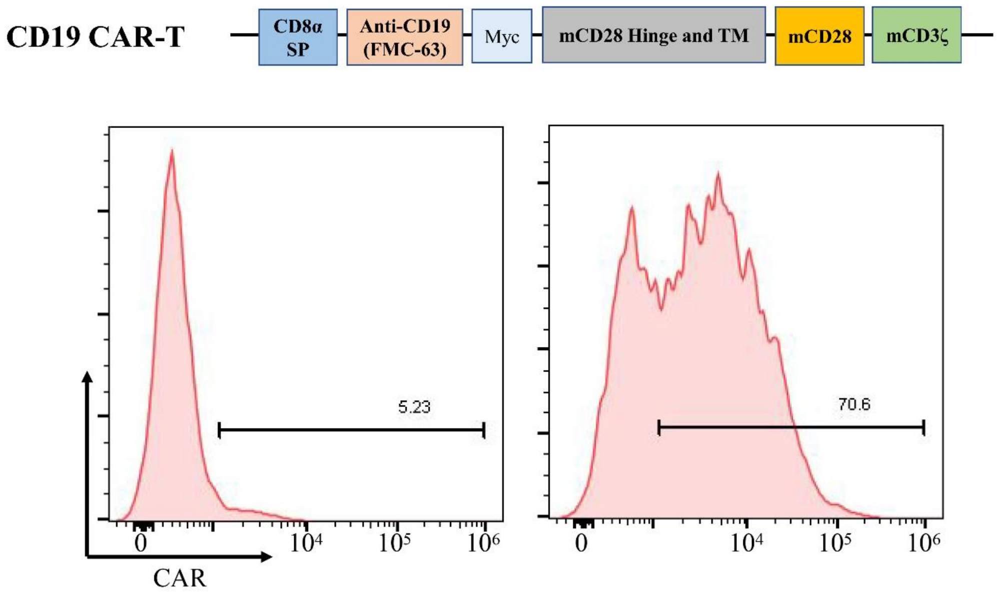

Figure S2. Construction and identification of murine CD19 CAR-T. (**A**) Schematic representation of retroviral vector expressing CD19 CAR construct. SP, signal peptide; TM, transmembrane domain. (**B**) Flow cytometric analysis of CAR expression on CAR-T cells.

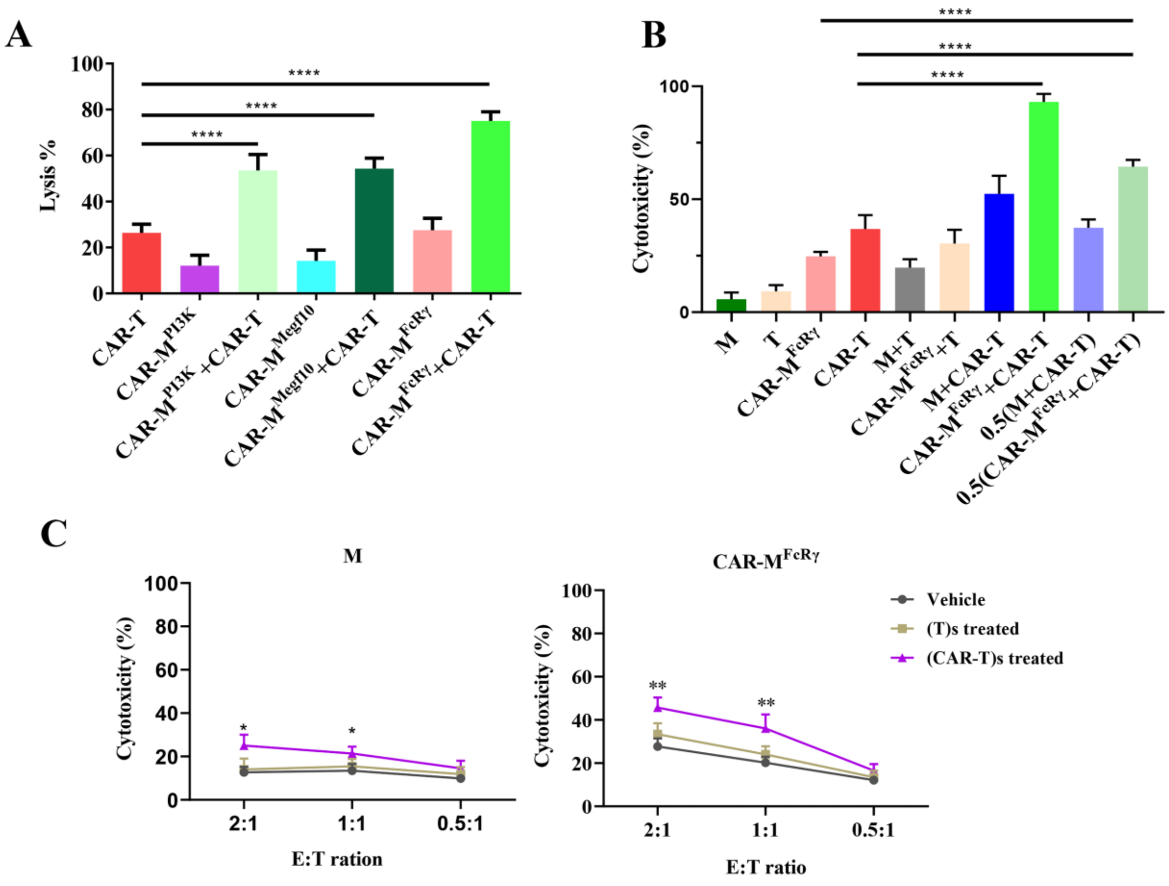

Figure S3. CAR-T and CAR-M demonstrated synergistic cytotoxicity against murine target cell Sp2/0-CD19+. (**A**) Luciferase-based cytotoxicity assay against Raji by CAR-T, CAR-Ms or the combination of CAR-M and CAR-T. The assay was performed after 48 h co-culture at an E:T ratio of 1 (CAR-T or CAR-Ms alone) or 2 (CAR-M + CAR-T). (**B**) Cytotoxicity assay against Sp2/0-CD19+ cells by different immune effector cells alone or different combination regimens after 48 h co-culture at an E:T ratio of 1 (M, T, CAR-M$^{FcR\gamma}$, CAR-T, 0.5 (CAR-M$^{FcR\gamma}$+CAR-T), 0.5 (CAR-M$^{FcR\gamma}$+CAR-T)) or 2 (M + T, CAR-M$^{FcR\gamma}$+T, M + CAR-T, CAR-M$^{FcR\gamma}$+CAR-T). M, GFP-M; T, untransduced T cells. (**C**) M or CAR-M$^{FcR\gamma}$ was treated by (T)s or (CAR-T)s for 24 h prior to cytotoxicity assay against Raji. Assays were performed after 48 h co-culture at different E:T ratios (2, 1 or 0.5). Data are represented as the mean ± SD of $n = 3$ biological replicates. Statistical significance was calculated using two-tailed Student t-test or one-way ANOVA analysis (*$p \leq 0.05$, **$p \leq 0.01$, ****$p \leq 0.0001$).

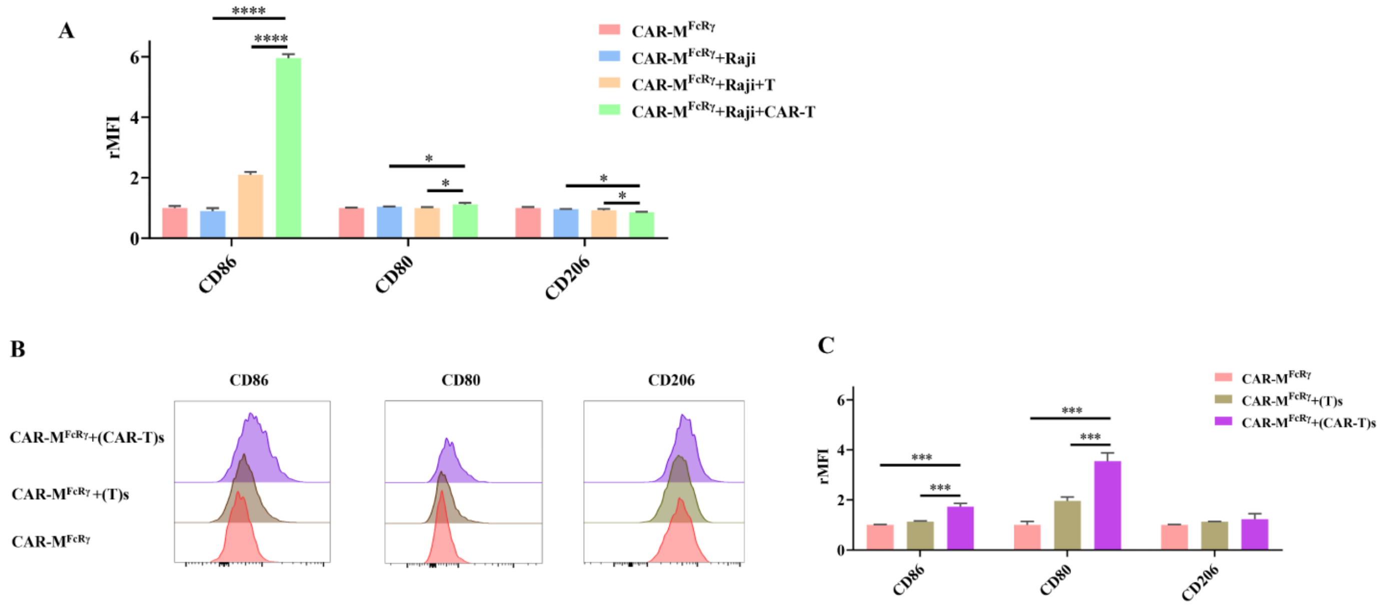

Figure S4. The polarization phenotype of CAR-M$^{FcR\gamma}$ from killing assay in combination with CAR-T or treated by the supernatants of CAR-T with Raji cells co-culture system. (**A**) Quantification of surface markers (CD86, CD80, CD206) expression of CAR-M$^{FcR\gamma}$ alone or co-cultured with Raji, Raji + T, Raji + CAR-T from (Figure. 4. D). Data are represented as the mean ± SD of n = 3 biological replicates. (**B**) Flow cytometric analysis of macrophage polarization M1 and M2 surface markers (CD86, CD80, CD206) of CAR-M$^{FcR\gamma}$ alone or treated by (T)s or (CAR-T)s for 24 h. The supernatants of T and CAR-T with Raji cells co-culture system after 48 h at an E:T ratio of 2 were referred to as (T)s and (CAR-T)s, respectively. (**C**) Quantification of surface markers (CD86, CD80, CD206) expression of CAR-M$^{FcR\gamma}$ alone CAR-M$^{FcR\gamma}$ alone or treated by (T)s or (CAR-T)s from panel (**B**). Data are represented as the mean ± SD of n = 3 biological replicates. rMFI, relative mean fluorescence intensity. Statistical significance was calculated using one-way ANOVA analysis (*$p \leq 0.05$, ***$p \leq 0.001$, ****$p \leq 0.0001$).

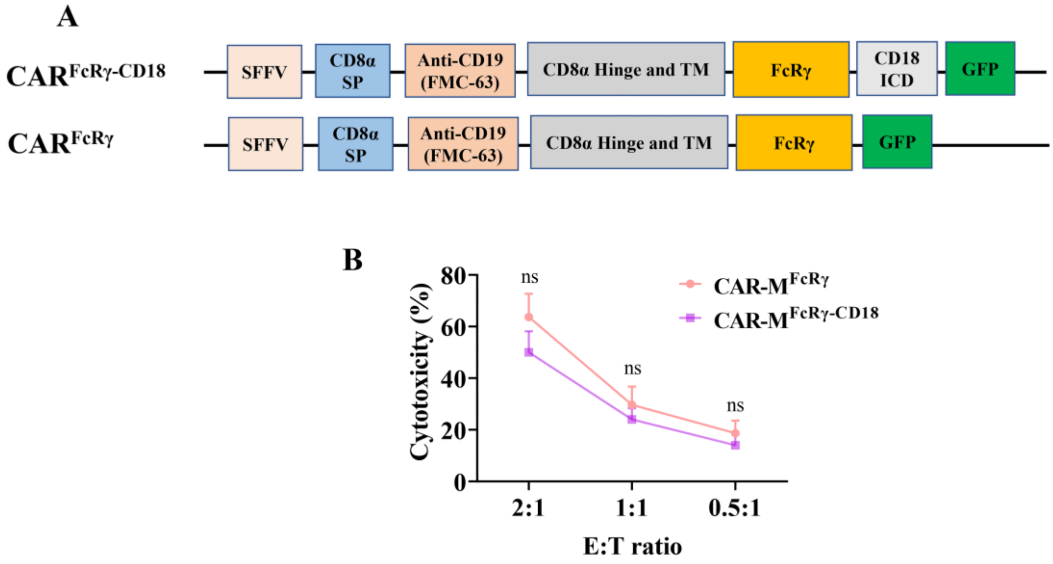

Figure S5. Comparation of cytotoxicity of CAR-M$^{FcR\gamma\text{-}CD18}$ and CAR-M$^{FcR\gamma}$. (**A**) Schematic representation of lentiviral vectors expressing CAR$^{FcR\gamma\text{-}CD18}$ and CAR$^{FcR\gamma}$. CD18 (aa 726-771 of Mouse CD18, Uniprot P11835). (**B**) Cytotoxicity assay against Raji by CAR-M$^{FcR\gamma\text{-}CD18}$ and CAR-M$^{FcR\gamma}$ after 48 h co-culture at different E:T ratios (2, 1 or 0.5). Data represent the mean ± SD of n = 3 biological replicates. Statistical significance was calculated using two-tailed Student t-test (ns, not significant).
